# Suicide bereavement in the UK: Descriptive findings from a national survey

**DOI:** 10.1111/sltb.12874

**Published:** 2022-05-25

**Authors:** Sharon McDonnell, Sandra Flynn, Jenny Shaw, Shirley Smith, Barry McGale, Isabelle M. Hunt

**Affiliations:** ^1^ Faculty of Biology, Medicine and Health Centre for Mental Health and Safety University of Manchester Manchester UK; ^2^ Suicide Bereavement Ramsbottom UK; ^3^ Greater Manchester Mental Health NHS Foundation Trust Manchester UK; ^4^ Independent Advisory Panel on Deaths in Custody London UK; ^5^ If U Care Share Foundation Chester UK; ^6^ Support After Suicide Partnership London UK

**Keywords:** bereavement, grief, online survey, postvention, suicide, support services

## Abstract

**Background:**

Those bereaved by suicide are a high‐risk group of adverse health outcomes and suicidal behavior, yet little is known about the experiences and support needs of these individuals in the UK.

**Methods:**

We conducted a national cross‐sectional study using an online survey and analyzed the experiences of 7158 participants who had been bereaved or affected by suicide.

**Results:**

Suicide had a major impact on 77% of participants, including those who had lost a friend and those exposed to suicide at a professional level. Mental and physical health problems linked to the suicide were reported in half. Adverse social outcomes and engaging in high‐risk behaviors following the suicide were common. Over a third reported suicidal ideation and 8% had attempted suicide as a direct result of the suicide loss. Most had not accessed support services, with the majority viewing provision of local suicide bereavement support as inadequate.

**Conclusions:**

Our results highlight the need for a multi‐disciplinary approach in postvention and the provision of proactive outreach to support those bereaved by suicide. Postvention efforts need to acknowledge the death of a friend by suicide as a significant loss.

## INTRODUCTION

Suicide is a major public health problem with over 700,000 people estimated to die by suicide globally each year (World Health Organization, 2019). The varying levels of impact suicide have on individuals who knew the deceased has been termed the “Continuum of Survivorship” by Cerel et al. (2014). This model suggests people can be “exposed” to suicide, that is, anyone who knows someone who has died by suicide, for example, first responders and acquaintances; others can be “affected” by suicide through experiencing distress but not regarding themselves as bereaved, and finally those who are termed the “suicide bereaved,” who experience significant short‐ or long‐term impact of the death. Those exposed to suicide, therefore, are not limited to close family members or friends, and estimations of suicide “survivors” range from 6 to 135 for every death by suicide (Berman, 2011; Cerel et al., 2018; Shneidman, 1973). With recent figures showing 6507 deaths registered as suicide in the UK in 2018 (Office for National Statistics, 2019a), this equates to potentially 39,000 to 878,000 people being impacted by suicide in the UK each year. This has important implications as adverse health outcomes are associated with suicide bereavement, albeit dependent on the closeness to the deceased (Mitchell et al., 2009). These include increased psychiatric morbidity, particularly depression, anxiety, and post‐traumatic stress disorder (Bolton et al., 2013; Erlangsen & Pitman, 2017; Mitchell & Terhorst, 2017), and physical illness such as cardiovascular disease (Spillane et al., 2017). Suicide bereavement also contributes to a higher risk of fatal and non‐fatal suicide attempt (Hamdan et al., 2020; Hill et al., 2020; Pitman et al., 2014; Pitman et al., 2016). Whilst much research has focussed on family members to the deceased, a review of the literature by Cerel et al. (2014) found 81.5% of studies reported an increased suicide risk in those exposed to suicide within non‐kin relationships. In a UK‐wide cross‐sectional survey, Pitman et al. (2016) found adults bereaved by suicide were 65% more likely to attempt suicide than those bereaved by sudden natural causes, irrespective of whether the deceased was blood‐related or not.

The increased risk of suicidal behavior among those exposed to suicide is, in part, explained by the prolonged bereavement reaction (also referred to as “complicated grief”) that can occur in those bereaved by suicide which can lead to acute psychological distress (Bellini et al., 2018). Severe grief reactions can result from the additional challenges faced by people who have experienced a death by suicide, such as intrusive thoughts and memories, trauma, perceived guilt, blame, and stigma (Bellini et al., 2018; Pitman et al., 2014; Young et al., 2012). Social stigma in particular is known to add burden to the bereavement process, with studies showing insensitivity from friends and family after a suicide death can lead to feelings of rejection and social isolation (Ross et al., 2021). Those bereaved are also known to receive less informal support from family and friends compared with those bereaved by other sudden death (Pitman et al., 2017). The experience of perceived or internalized stigma can also reduce help‐seeking behavior which in turn may increase the risk of suicidal behavior (Carpiniello & Pinna, 2017).

Whilst much research on suicide bereavement relates to family members, exposure to suicide in occupational settings has also been described, particularly among emergency services including the police and ambulance staff (Cerel, Jones et al., 2019; Nelson et al., 2020) but also among mental health, educational and social care personnel (Awenat et al., 2017; Causer et al., 2019). Despite the professional relationship these groups have with the deceased, there is growing evidence that occupational exposure to suicide is associated with significant emotional trauma and poor mental health (Aldrich & Cerel, 2020; Lyra et al., 2021), particularly for those with multiple exposure to suicide (Nelson et al., 2020).

Acknowledgement of the far‐reaching effect of suicide is reflected in the substantial increase in suicide bereavement research over the past two decades (Maple et al., 2018). This has led to policy initiatives that address specific care provision, that is, postvention, for those bereaved after suicide (NHS Long Term Plan, 2019). A number of national suicide prevention strategies globally now include postvention as a key objective in addressing the needs of those bereaved by suicide (WHO, 2018). In England, these include commitments within the NHS Long Term Plan (2019) to ensure post‐crisis support is available to families and staff working in mental health crisis services who have been bereaved by suicide. Despite the increased attention suicide bereavement is garnering from academics and policy makers, the effectiveness of interventions for prolonged grief have yet to be determined (Andriessen, Krysinska, Hill et al., 2019; Andriessen, Krysinska, Kolves et al., 2019). A recent qualitative study reported parents who had lost a child to suicide felt there were barriers in accessing support in primary care with general practitioners being unaware of where to signpost for support (Wainwright et al., 2020). These findings confirm other qualitative work that those bereaved by suicide do not always receive the necessary help and support they need (Andriessen et al., 2020; Pitman et al., 2014; Ross et al., 2021).

Further knowledge is required to enhance our understanding of suicide bereavement and examine views on available support services. Previous studies have varied in settings, populations and sample sizes (Azorina et al., 2019; Cerel, Jones et al., 2019; Ross et al., 2021), and few national studies have been carried out to examine the impact of suicide at a personal and professional level (Maple et al., 2019; Pitman et al., 2016). Our aims, therefore, were firstly to understand the impact a death by suicide had on those who were bereaved or affected, and secondly to examine whether support services or resources were available and offered after a death by suicide, whether these were utilized and what was viewed as helpful.

## METHODS

### Study design and questionnaire

A UK‐based cross‐sectional study was undertaken. An online survey was initially developed by the authors, with survey questions informed by some authors' lived‐experience and from existing literature. The survey was refined after piloting with a group of experts from bereavement services including a patient and public involvement (PPI) group. The survey was administered through an online survey platform “SelectSurvey” and was open between July 2017 and August 2018. The survey included 71 questions, predominantly checkbox questions but some free‐text questions to elicit more details on the experiences of being bereaved or affected by suicide. Findings related to the qualitative sections of the survey will be reported elsewhere. The participant information sheet provided broad definitions of being bereaved by suicide (personally bereaved e.g., loss of a family member, friend, and colleague) or affected by suicide (not personally bereaved e.g., frontline staff, neighbor, and prison officers). Sections of the survey covered socio‐demographics; suicide in the workplace; general details of the suicide (e.g., relationship and timing); the impact of suicide (e.g., adverse social and health factors, high‐risk behaviors); and support services (e.g., whether support was offered or accessed and views on their experience). All participants were advised to refrain from answering any questions found to be too sensitive, if they became distressed. Where participants had experienced multiple suicides, we asked them to provide responses based on one death by suicide. This was to enable the research team to understand the totality of the lived experience of one single suicide and avoid examination of the effects of multiple exposures to suicide. The full survey is available from the corresponding author.

### Participants

Inclusion criteria were those aged 18 and above; resident in the UK; and who perceived themselves as bereaved or affected by suicide. Participants were recruited via social media platforms (e.g., Twitter, Facebook); authors' affiliation websites (e.g., the Centre for Mental Health and Safety, University of Manchester, and the Support After Suicide Partnership); advertising flyers via newspapers, radio, and TV; academic conferences; and by word of mouth.

A total of 9744 people opened the online questionnaire, 1699 (17%) of whom did not answer any of the survey questions. Of the remaining 8045, 887 (11%) were excluded due to missing data (*n* = 630) or not meeting the eligibility criteria, i.e., being aged under 18 (*n* = 64) or living outside of the UK (*n* = 193).

### Study procedure

The survey was completed online or a paper version was available for those without internet access. Participants provided electronic consent prior to beginning the survey (or written consent for those completing a paper version). All responses were anonymous. At the end of the survey, participants were directed to a website listing details of key support resources and contact details of suicide bereavement organizations to minimize any potential distress. No compensation was offered for participation in the study and respondents could discontinue the survey at any point. The study was approved by the University of Manchester Research Governance and Ethics committee (ref: 14432, Research Ethics Committee 3, 28th May, 2015).

### Statistical analysis

Descriptive statistics (frequencies and percentages) were used for demographic and categorical data. Pearson's chi‐square tests for differences in proportions were used for pairwise comparisons. We removed cases where data were not known for that item. Statistical tests were two sided, with *p* < 0.05 interpreted as statistically significant. Stata 15.0 was used for all statistical analysis (StataCorp, 2017).

## RESULTS

In total, 7158 people responded to the sections on being bereaved or affected by suicide and these represented our final sample. Responses were received across the UK: 84% from England, 7% Scotland, 5% Wales, 3% Northern Ireland, and <1% from the Isle of Man and the Channel Islands.

### Participant characteristics

Table 1 shows the socio‐demographic characteristics of the respondents. The majority were female (78.7%) and ranged in age from 18 to 84 years (mean, 43.6, SD = 13.0). Three percent were from ethnic minority backgrounds, lower than the proportion of ethnic minority groups in the UK (14%, ONS, 2019b). The majority (89%) identified as heterosexual. Those who identified as non‐heterosexual were significantly younger than other respondents (mean age 35 vs. 44; *p* < 0.001). For a fifth (1229, 21%) of respondents the suicide had occurred less than a year ago, 2051 (36%) between 1 and 5 years ago, 891 (15%) over 5 but <10 years ago, 883 (15%) between 10 and 20 years ago, and 702 (12%) over 20 years ago. Three‐quarters (75%) were in full‐time employment or self‐employed. Of the employed responders, 2318 (43%) were categorized as professionals; 788 (15%) were managers, directors and senior officials; 698 (13%) were administrative and secretarial; and 691 (13%) were in caring and leisure occupations.

**TABLE 1:** Socio‐demographic characteristics of survey respondents

Characteristic	All *n* = 7158 *n* (%)	Male *n* = 1519 *n* (%)	Female *n* = 5627 *n* (%)	*p*‐Value^*^
Age (years)	*n* = 7004	*n* = 1490	*n* = 5503	
18–24	625 (8.9%)	128 (8.6%)	492 (8.9%)	0.007
25–44	2919 (41.7%)	673 (45.2%)	2241 (40.7%)	
45–64	3114 (44.5%)	631 (42.4%)	2482 (45.1%)	
65+	346 (4.9%)	58 (3.9%)	288 (5.2%)	
Living circumstances	*n* = 7119	*n* = 1507	*n* = 5600	
Alone	1204 (16.9%)	255 (16.9%)	942 (16.8%)	<0.001
With parent(s)	546 (7.7%)	152 (10.1%)	391 (7.0%)	
With spouse/partner	4168 (58.6%)	955 (63.4%)	3211 (57.3%)	
With child(ren) only	773 (10.9%)	40 (2.7%)	733 (13.1%)	
Other	428 (6.0%)	105 (7.0%)	323 (5.8%)	
Ethnicity	*n* = 7137	*n* = 1518	*n* = 5619	
White	6910 (96.8%)	1469 (96.8%)	5441 (96.8%)	0.277
Asian/Asian British	73 (1.0%)	11 (0.7%)	62 (1.1%)	
Black/Black British	32 (0.5%)	9 (0.6%)	23 (0.4%)	
Mixed/multiple ethnicity	106 (1.5%)	23 (1.5%)	83 (1.5%)	
Other	16 (0.2%)	6 (0.4%)	10 (0.2%)	
Employment status	*n* = 7113	*n* = 1509	*n* = 5604	
Employed/self‐employed	5347 (75.1%)	1216 (80.6%)	4127 (73.6%)	<0.001
Unemployed	271 (3.8%)	58 (3.8%)	213 (3.8%)	
Full‐time student	398 (5.6%)	71 (4.7%)	327 (5.8%)	
Housewife/husband	254 (3.6%)	5 (0.3%)	249 (4.4%)	
Long‐term sick leave	257 (3.6%)	42 (2.8%)	215 (3.8%)	
Retired	566 (8.0%)	111 (7.4%)	455 (8.1%)	
Other	24 (0.3%)	6 (0.4%)	18 (0.3%)	
Sexual orientation	*n* = 7118	*n* = 1512	*n* = 5606	
Straight/heterosexual	6337 (89.0%)	1306 (86.4%)	5031 (89.7%)	<0.001
Gay/homosexual	261 (3.7%)	117 (7.7%)	144 (2.6%)	
Bisexual	318 (4.5%)	57 (3.8%)	261 (4.7%)	
Pansexual	39 (0.6%)	7 (0.5%)	32 (0.6%)	
Other	27 (0.4%)	7 (0.5%)	20 (0.4%)	
Prefer not to say	136 (1.9%)	18 (1.2%)	118 (2.1%)	

^*^ *p*‐Values for group comparisons excluding missing values.

### Frequency of exposure to suicide

Two thirds (4816, 67%) of respondents reported being bereaved or affected by one single suicide, while a third (2342, 33%) had experienced more than one suicide, ranging from 2 to 70 deaths. Four hundred and seventy seven (7%) respondents experienced between four and 70 deaths by suicide. Exposure to high numbers of deaths by suicide was common among occupations such as health professionals (99, 26%), caring personal services (i.e., care workers, nursing auxiliaries and assistants; 43, 11%), and protective services (i.e., police, firefighters, prison officers; 35, 9%). The occupation with the highest exposure to suicide was crime scene examiner. Participants from Northern Ireland were more likely than those from other UK countries to have experienced more than one suicide (44% vs. 32%).

### Relationship to the deceased

**FIGURE 1:**
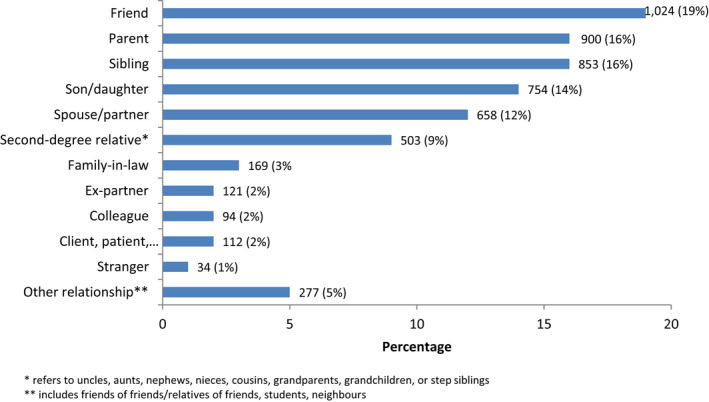
Relationship of the participants to the individual who died by suicide

In total, 5499 participants provided information on their relationship to a person who had died by suicide. The most common relationship was the death of a friend (19%), followed by a parent (16%), sibling (16%), or a son/daughter (14%) (Figure 1). There were 206 (4%) respondents who reported the death to be someone known through their occupation (i.e., a colleague or client).

### Impact of the suicide and adverse life events

The majority (77%) of respondents reported the suicide had a major impact on their lives, particularly those who had lost a family member (95%). Only 20 (<1%) reported no impact from the death by suicide. Adverse social life events following the death by suicide were reported by over a third (39%) of respondents. The most common were family problems, relationship breakdown, and financial difficulties (Table 2). Women were more likely to have reported adverse events compared to men, especially family problems, unemployment/job loss, and financial problems. Gambling was more commonly reported by men than women.

**TABLE 2:** Self‐reported adverse social and health‐related events and suicidal behavior following the death by suicide

Event	All *n* = 7158 *n* (%)	Male *n* = 1519 *n* (%)	Female *n* = 5627 *n* (%)	*p*‐Value
Any social life events	2817 (39)	480 (32)	2328 (41)	<0.001
Family problems^a^	1671 (23)	271 (18)	1395 (25)	<0.001
Relationship problems^a^	1306 (18)	283 (19)	1019 (18)	0.640
Financial problems	905 (13)	129 (8)	772 (14)	<0.001
Moved home	862 (12)	136 (9)	721 (13)	<0.001
Unemployment/job loss	558 (8)	96 (6)	458 (8)	0.019
Divorce/break‐up	458 (6)	99 (7)	357 (6)	0.807
Homelessness	161 (2)	29 (2)	128 (2)	0.388
Gambling^a^	60 (1)	36 (2)	24 (<1)	<0.001
Any health events	3482 (49)	636 (42)	2835 (50)	<0.001
Mental health problems	2629 (37)	484 (32)	2135 (38)	<0.001
Poor physical health^a^	1550 (22)	237 (16)	1309 (23)	<0.001
Alcohol use^a^	1285 (18)	316 (21)	964 (17)	0.001
Use of prescription drugs	1041 (15)	144 (9)	892 (16)	<0.001
Self‐harm	543 (8)	81 (5)	455 (8)	<0.001
Illicit drug misuse^a^	378 (5)	138 (9)	239 (4)	<0.001
Hospitalization for mental health problems	203 (3)	39 (3)	161 (3)	0.538
Suicidal behavior				
Suicidal ideation	1911/5056 (38)	350/979 (36)	1551/4066 (38)	0.303
Suicide attempt	382/4818 (8)	82/953 (9)	294/3854 (8)	0.456

^a^ Experienced for longer than 3 months.

**FIGURE 2:**
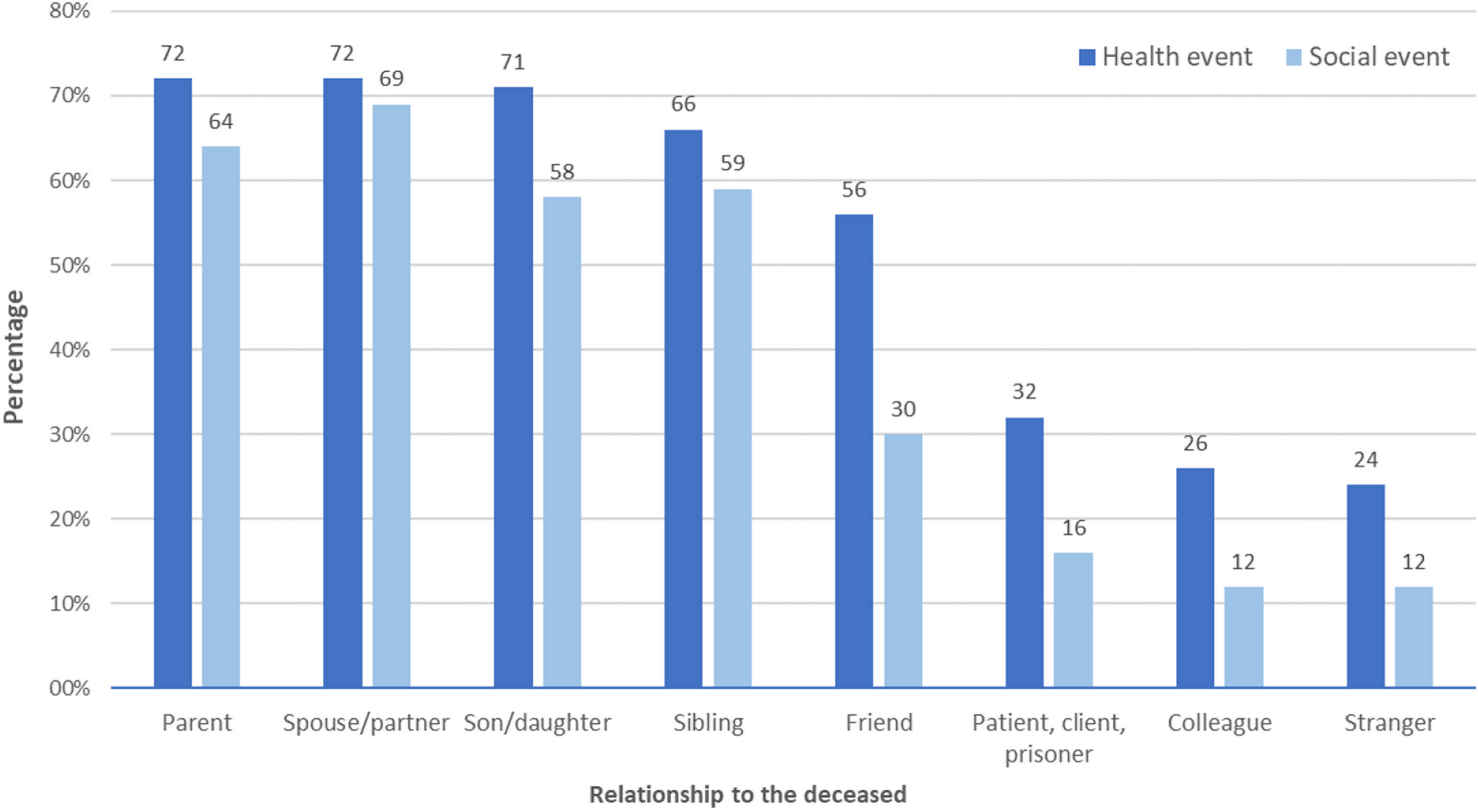
Adverse health‐related and social life events following the suicide by relationship to the deceased

Health‐related problems judged by participants to be linked to the death by suicide were reported by half of the sample. These were more often reported by women than men, particularly mental illness, deterioration in physical health, and use of prescription drugs (Table 2). Self‐harm was also more often reported in women and in those aged under 25 compared with older respondents (145, 23% vs. 390, 6%). In contrast, illicit drug use and alcohol use were more often reported by men. Mental health problems were reported by 37% of participants, most commonly by those closely related (parent, child, and sibling) to the deceased (1280, 51%) or a spouse/partner (336, 51%) but also by those who had lost a friend (475, 46%). Examples of the self‐reported mental health problems included anxiety and panic disorders, post‐traumatic stress disorder, depression, and eating disorders. Multiple health‐related problems were common, with 2233 (31%) reported experiencing two or more health issues, and 1394 (19%) three or more. Whilst adverse health‐related and social life events (e.g., relationship breakdown) were more prevalent among participants related to, or friends with, the deceased, they were also reported in around a quarter of those affected by a suicide in an occupational setting, that is, a clients, patients, or colleagues, and among those exposed to a death of a stranger (Figure 2).

### High‐risk behaviors

Of 5470 respondents, 1641 (30%) reported they had engaged in high‐risk behaviors following the suicide, 1055 of whom provided specific details. The most common related to alcohol and drug misuse (494, 47%), recklessness with finances (259, 25%), sexual promiscuity (193, 18%), lack of road safety (187, 18%), and aggressive behavior (118, 11%). Men more often reported high‐risk behaviors (363, 33% vs. 1271, 29%; *p* = 0.006) as did those aged under 25 (199, 47% vs. 1415, 29%; *p* < 0.001). Differences between risk behaviors and the personal relation to the deceased were observed, with those bereaved by a parent (300, 21% vs. 517, 14%; *p* < 0.001) or a spouse/partner (240, 17% vs. 382, 11%; *p* < 0.001) more likely to have partaken in high‐risk activities. Those who had been bereaved or affected by suicide for longer than a year were significantly more likely than other participants to have engaged in high‐risk behaviors (1240, 82% vs. 2933, 77%; *p* < 0.001).

### Suicidal behavior

Of 5056 respondents, 1911 (38%) reported suicidal ideation and 382 (8%) of 4818 respondents made a suicide attempt following the person's death. These proportions were similar for men and women (Table 2). The most common relationships to the deceased in those who reported a suicide attempt were: parent (83, 23%); friend (80, 22%); spouse/partner (66, 19%); sibling (47, 13%); and child (40, 11%). The majority (229, 64%) had attempted suicide within a year following the death by suicide; 28 (12%) of whom attempted suicide within the first week after the loss.

### Access to support services

Forty percent (*n* = 2864) of participants had accessed support from one or more services following the death by suicide. This was most commonly from their GP (1164, 16%), private counseling/bereavement support (966, 14%), or online support (957, 13%), but also included suicide bereavement services (639, 9%), self‐help groups (491, 7%), and information leaflets (457, 6%). Most (1116 of 1732 respondents, 65%) reported their preferred time for being approached and offered support was within a week of the death; a quarter (433, 25%) preferred to be approached between 1 week and 1 month after the suicide. Of 808 respondents who provided information on support provided by their employer, around half (458, 53%) had been offered support.

Reasons for not accessing support were provided by 1575 participants, and included having supportive families and friends (624, 40%), feeling able to cope alone (576, 37%), and being unaware of available services (557, 35%). Twelve percent (*n* = 192) reported there were no local support services available to them. Overall, the provision of suicide bereavement support in the local area was viewed as inadequate by 2876 (62%) of 4621 respondents.

## DISCUSSION

### Main findings

Our results have provided an overview of the impact a death by suicide can have on those bereaved. We found participants reported serious psychological and physical health problems, including suicidal behavior, and perceived these had occurred as a consequence of being exposed to suicide. Adverse outcomes were present not only among those related to the deceased but also for those who had lost a friend to suicide or experienced suicide in an occupational setting. Participants described a lack of accessing support services, with the majority reporting provision of local suicide bereavement support to be inadequate.

### Findings in relation to previous work

Little research has examined suicide bereavement specifically among non‐kin relationships yet friends are also known to be vulnerable to the detrimental outcomes following a suicide (Bartik et al., 2013; Cerel et al., 2014; Pitman et al., 2016). A large longitudinal survey in Australia noted the death of a close friend (not specifically by suicide) was a substantial experience and led to poorer mental health and social functioning up to 4 years after the bereavement (Liu et al., 2019). The unique challenges faced by the suicide‐bereaved can increase the likelihood of disenfranchized or complicated grief, and this has particularly been reported among young adult friends of the deceased (Prigerson et al., 1999).

Our finding of suicide impacting on physical health is in keeping with previous work showing an increased risk of major physical illnesses in those bereaved by suicide (Bolton et al., 2013). However, a systematic review by Spillane et al. (2017) revealed mixed results on the association between suicide bereavement and adverse physical health outcomes and concluded further research is needed to fully understand this relationship. Clearer evidence exists for an association between suicide bereavement and adverse mental health outcomes, including depression and anxiety disorders, and hospital admission to psychiatric care (Pitman et al., 2014; Cerel et al., 2016; Pitman et al., 2016; Bolton et al., 2013; Omerov et al., 2013). The current study supports this with over a third reporting mental health problems following the death by suicide, particularly by those experiencing the loss of a familial or spousal relationship but also by friends. Despite reporting mental and physical health problems, the majority had not accessed support from services, but instead felt adequate support was provided from family and friends, consistent with previous research (Provini et al., 2000; Spillane et al., 2017).

Over a third of our respondents had reported suicidal ideation, a higher proportion than a recent Australian study by Maple et al. (2019) who found 18.5% of people exposed to suicide reporting suicidal thoughts, although this was experienced over the previous year whilst our data did not specify a time frame. Our finding that high‐risk behaviors such as self‐harm and suicide attempt were features among people aged under 25 corroborates those of Wilcox et al. (2010) who found that following the suicide of a close friend or relative, young people had an increased risk of suicide attempts or hospitalization due to mental illness. Pitman et al. (2016) also reported an association between exposure to suicide and subsequent suicide attempt among young adults, though this effect became non‐significant when adjusting for perceived stigma. Unlike the typical gender gap where more women than men attempt suicide (De Jong et al., 2010), we found a similar proportion reported a suicide attempt post‐bereavement. A comparable excess risk of self‐harm in different gender groups has previously been reported after a spousal suicide (Erlangsen & Pitman, 2017) and highlights the importance of encouraging people to seek support following a bereavement.

Previous research has shown adolescents exposed to peer suicidal behavior engage in high‐risk behaviors such as substance misuse, physical fights, and aggression (Bartik et al., 2013; Cerel et al., 2005), and our results are consistent with this in an adult population. Such behavioral indicators of grief have been associated with complicated grief, known to disrupt functioning (Shear, 2012). We also found these respondents were more likely to exhibit such behaviors more than a year after the death, suggesting the length of time since the suicide did not lessen the impact of the grief for some individuals (Cerel et al., 2005). To mitigate the detrimental effects of a suicide bereavement, access to support should therefore be available long term and be easily accessible. This has been highlighted by Wilson and Marshall (2010) who found over a quarter of bereaved participants required help from a professional for 12 months following the suicide and a fifth for at least 2 years.

Few respondents in the current study were aware of suicide bereavement services, particularly local services, and the majority viewed provision of support as inadequate.

Given the difficulties participants reported in identifying bereavement support services, a proactive support model should facilitate timely access to essential information and care pathways. International experience suggests that “active postvention” is needed where support is brought to those bereaved rather than the bereaved finding support themselves (Cerel & Campbell, 2008; Ross et al., 2018). Expanding the reach of services to those outside of the deceased's immediate family is also advocated, particularly as individuals who have lost a friend to suicide are known to suffer mental distress that will go untreated (Feigelman et al., 2019).

### Strengths and limitations

A key strength of the study includes the large national population‐based sample, which is the largest till date in suicide bereavement research. The use of a population‐based survey avoids the biases associated with collecting data using help‐seeking samples. We also obtained information from over 1500 men who are traditionally recognized as a hard to reach group, particularly in relation to discussing grief (Pitman et al., 2014). Similarly, we achieved large samples of LGBT and ethnic minority communities bereaved or affected by suicide. However, the results should be considered in light of certain limitations. The cross‐sectional design means we cannot establish causality. Those who chose to take part in the study may have been more affected by the death by suicide than those who did not, thereby potentially over estimating the prevalence of adverse outcomes. This “self‐selection” bias is common in online surveys and means some groups are under‐represented, for example, those who do not have access to the Internet. We are therefore unable to generalize the findings to all those bereaved or affected by suicide, and our age criteria (18 and above) means we cannot establish the grief experiences of young people, but this will be an important area for future study. We did not use any standardized measures for outcomes, thus limiting comparison with previous studies. In addition, the level of missing data was high for some survey items and we are unable to determine whether those who did not respond to particular questions differed from those who responded (“nonresponse bias”). Adverse physical and mental symptoms were self‐reported and could not be validated with standardized measures or health records. In addition, we were not able to determine whether participants had existing premorbid conditions, such as depression or suicidal ideation, which were not attributed to the bereavement. Our findings relate to participants' experiences following one death by suicide, and we are unable to determine the confounding effects of multiple exposures to suicide, which warrants further study. Finally, the retrospective nature of the study may have introduced recall bias, particularly for those exposed to the suicide decades previously.

### Implications

We have highlighted the varied impact of suicide and the clear need for those bereaved or affected by suicide receive a timely and co‐ordinated response by services. Positive advances are underway with suicide bereavement support services being rolled out nationwide in the UK (NHS Long Term Plan, 2019). However, whether these services are consistent in delivering high‐quality bereavement support remains to be seen and requires monitoring. Our key recommendation is the implementation of national minimum standards in postvention services. These include a multi‐agency, holistic approach which focuses on practical help such as financial advice, as well as support for physical health, substance misuse, and psychological wellbeing. Provision of practical support and guidance could take many forms including helplines, support groups, literature, and counseling, but should be consistent across the NHS and third sector organizations. Providing a wide range of quality resources would make services more inclusive. Mental health and postvention services should recognize the vulnerabilities in friends who have lost someone to suicide and address any associated disenfranchised grief. However, this requires the recognition that impact should not be “perceived” on the basis of kinship, but on psychological closeness (Cerel et al., 2013; Maple et al., 2016).

Staff working in any postvention service (NHS or third sector) should be sufficiently trained and equipped with the skills to recognize and address the complex grief, trauma, and adverse behavioral responses associated with suicide bereavement. Other agencies likely to be in contact with people recently bereaved by suicide including GPs, frontline staff, funeral directors, and social support services, also need the knowledge to signpost support effectively. This is in line with the quality standard on suicide prevention by the UK National Institute for Health and Care Excellence (NICE, 2019) which acknowledges those bereaved or affected by suicide are at increased risk of suicide themselves.

In addition to responding to immediate support needs, ongoing multi‐agency care and proactive outreach should be provided to ensure risk can be safely managed over time. Interventions such as the Australian “StandBy” response service, which provides immediate support and clear pathways to care for people impacted by suicide, has been shown to reduce levels of suicidality and social isolation associated with suicide bereavement (Gehrmann et al., 2020). Such programs have been widened to include those affected by suicide in the workplace. Recent advances by Business in the Community (2019) in association with Public Health England and the Samaritans include the development of a suicide prevention toolkit as part of a strategy to reduce the risk of workplace suicide.

### Directions for future research

The differential experiences of those who have been affected or bereaved by suicide suggests interventions need to be multi‐dimensional, but more research is needed to ascertain the most effective methods of delivering support to different groups. A number of core standards have recently been recommended by the Support After Suicide Partnership to inform services and ensure effective evidence‐based support can be delivered to those bereaved (https://hub.supportaftersuicide.org.uk/standards/). Future service development needs to consider the recommendations of those with lived experience and recognize the prolonged levels of distress that can occur from this unique grief. A multi‐agency approach combining efforts from voluntary sector organizations, mental and physical healthcare providers, social care, and community services could help develop a sustainable model of resourcing at local, regional, and national levels. Ultimately, developing tailored evidence‐based programs for all people affected or bereaved by suicide may help to reduce the short‐ and long‐term negative outcomes following a death by suicide. However, a comprehensive research agenda is required to monitor the standards and ensure services deliver equitable postvention support.

## CONFLICT OF INTEREST

The authors have no conflicts of interest to declare.

## ETHICAL APPROVAL

Ethics approval for the study was obtained from the University of Manchester Research Ethics committee (reference number: 2017‐1818‐2599).

## Data Availability

Research data are not shared.
